# Selenium level and depressive symptoms in a rural elderly Chinese cohort

**DOI:** 10.1186/1471-244X-12-72

**Published:** 2012-07-03

**Authors:** Sujuan Gao, Yinlong Jin, Frederick W Unverzagt, Chaoke Liang, Kathleen S Hall, Jingxiang Cao, Feng Ma, Jill R Murrell, Yibin Cheng, Ping Li, Jianchao Bian, Hugh C Hendrie

**Affiliations:** 1 Department of Biostatistics, Indiana University School of Medicine, Indianapolis, IN, USA; 2 Institute for Environmental Health and Related Product Safety, Chinese Center for Disease Control and Prevention, Beijing, China; 3 Department of Psychiatry, Indiana University School of Medicine, Indianapolis, IN, USA; 4 Department of Pathology and Laboratory Medicine, Indiana University School of Medicine, Indianapolis, IN, USA; 5 Sichuan Provincial Center for Disease Control and Prevention in China, Chengdu, China; 6 Shandong Institute for Prevention and Treatment of Endemic Disease in China, Jinan, China; 7 Indiana University Center for Aging Research, Indianapolis, IN, USA; 8 Regenstrief Institute, Inc., Indianapolis, IN, USA; 9 Department of Biostatistics, Indiana University School of Medicine, 410 West 10th Street, Suite 3000, Indianapolis, IN, 46202-2872, USA

## Abstract

**Background:**

Selenium is considered a protective agent against free radicals through the maintenance of better enzyme activity. The few studies examining the relationship between selenium and depression have yielded inconsistent results and none of these studies considered the role of cognitive function in this context.

**Methods:**

A cross-sectional evaluation of 1737 rural Chinese age 65 and over from two provinces in China was conducted. Depressive symptoms were assessed using the Geriatric Depression Scale (GDS). Cognitive function was assessed using various cognitive instruments. Selenium measures were obtained from nail samples. Other information collected included demographic characteristics and medical history. Analysis of covariance models were used to identify factors associated with GDS score.

**Results:**

Higher selenium levels were associated with lower GDS scores adjusting for demographic and medical conditions (p = 0.0321). However, the association between selenium and depressive symptoms was no longer significant when cognitive function score was adjusted in the model (p = 0.2143).

**Conclusions:**

Higher selenium level was associated with lower depressive symptoms without adjusting for cognition in this cohort. However, after cognition was adjusted in the model the association between selenium and depressive symptoms was no longer significant, suggesting that selenium’s association with depressive symptoms may be primarily through its association with cognitive function.

## Background

Selenium is a trace element associated with the antioxidant enzyme glutathione peroxidase (GP-x) and is considered a protective agent against free radicals through the maintenance of better enzyme activity. Several studies examining the relationship between selenium and depression have provided inconsistent results, with some showing lower selenium levels associated with greater risk of depression [1-4] while others showing no association [5]. It is interesting to observe that a beneficial effect of selenium supplementation was mostly seen when a substantial number of participants had low dietary selenium levels and selenium supplementation in subjects with already adequate selenium levels does not seem to confer benefit on mood or depressive symptoms [1-3]. It is also worth noting that none of these studies considered cognitive function of the study participants.

Research has shown that the brain has a unique feature in selenium metabolism by storing selenium so that GP-x activity in the brain does not decrease as fast as in the liver after a low selenium diet [6,7]. Based on this observation, it has been hypothesized that long-term exposure to low selenium may be needed to impact brain function such as mood or cognitive function [8]. Given that selenium content in food, especially grain, is highly variable depending on selenium content of the soils in which they are grown [9], studying the relationship between long-term selenium exposure and brain functions may be difficult in populations that are mobile and consume foods produced in different areas of the world. Moreover, supplements containing selenium are often ingested particularly by health conscious individuals thus confounding study results.

The rural elderly Chinese population represents an opportunity for examining the relationship between long-term selenium exposure and depressive symptoms. The rural Chinese are stable with most living in the same village throughout their entire life and consuming locally grown food. In addition, it is rare for them to take dietary supplements. Our group has previously reported on the association between selenium and cognitive function in this cohort. In this article, we investigate the association between selenium levels and depressive symptoms and examine the role of cognitive function on the association between selenium and depression in the same cohort.

## Methods

### Study population

A total of 2000 older adults were recruited for the Selenium and Cognitive Decline study, a longitudinal epidemiologic project funded by the National Institute of Health examining the long-term impact of selenium on cognitive function in rural elderly Chinese [8]. Two counties from Sichuan Province in southwestern China and two counties from Shandong Province in eastern China were selected based on: rural areas with historically low in- and out-migration, wide range of selenium levels but comparable trace elements otherwise, comparable age and gender proportions, and sufficient population to provide 500 elderly subjects per county. Sites with known endemic diseases (including Keshan disease, Kaschin-Beck disease, goiter and Cretinism, and fluorosis) were excluded from consideration.

For each village included in the study, a team of interviewers who were employees from provincial or county offices of Chinese Center for Disease Control traveled to the area, established a temporary headquarter and conducted a complete census of residents over age 65 in the area. They enrolled eligible residents by going door-to-door, obtaining informed consent before conducting the interview and collecting biological samples. There were no refusals. However, a few subjects with hearing problems were not enrolled. The study was approved by Indiana University Institutional Review Board and the Institute for Environmental Health and Related Safety, Chinese Center for Disease Control and Prevention.

A second evaluation of this cohort was conducted approximately two and half years after the initial evaluation. In addition to cognitive assessment, the Geriatric Depression Scale (GDS) was administered to all participants during the second evaluation.

### Measure of depressive symptoms

The GDS is a 30-item scale developed specifically for use in elderly populations [10]. GDS scores between 11 and 20 are generally considered to represent significant mild depression and scores of 21 or higher are considered severe depression [11]. The GDS was validated with a psychiatric outpatient sample in a previous study in Chinese age 60 or older [12]. Internal consistency was high (alpha = 0.89), and the test-retest reliability was 0.85. The GDS also showed excellent criterion-related validity (0.95) when compared to psychiatrist diagnosis, and substantial concurrent validity (0.96) when compared with the Center for Epidemiologic Studies Depression Scale (CES-D) [13]. Although the GDS is generally self administered, because of low literacy rates in our cohort, the GDS was administered by trained interviewers in face-to-face interviews.

### Selenium measures

Nail samples from all study subjects were collected during the initial interview and stored in clean plastic bags labeled with participant identification numbers. The method of fluorometric determination of trace amount of selenium with 2,3-diaminonaphthalene, described in details elsewhere [14], was used to determine trace amounts of selenium in nail samples. Quality control in the laboratory was maintained by using certified reference materials and by inter-laboratory comparisons. Quality control measures used for the laboratory analyses were described in details previously [8].

### Cognitive assessment

Cognitive assessment was conducted in face-to-face interviews using the Community Screening Instrument for Dementia (CSID), Consortium to Establish a Registry for Alzheimer’s Disease (CERAD) 10-word list learning and recall,[15] Indiana University (IU) Story Recall, Animal Fluency test [16], and IU Token test at baseline and at the 2.5 year follow-up. The CSID was developed as a screening tool for dementia in populations with various cultural backgrounds and literacy levels. The CSID has demonstrated good two week test retest reliability and inter-rater reliability as well as good validity in detecting dementia in various populations [17,18]. CSID scores range from 0 to 30. The CERAD Word List Learning test is one of the measures from the CERAD neuropsychological assessment battery which was designed to assess cognitive skills in the elderly. The IU Story Recall task was created by the research team to be suitable to the Chinese culture and the rural population. The Animal Fluency test is a measure of executive function in which a subject names as many animals as possible in 60 seconds. The IU Token Test is a brief measure of language comprehension and working memory [19]. The validity of the CSID, CERAD word list learning and recall, and the Animal Fluency test have been previously established in Chinese population and elsewhere [20].

### Other information

Other information collected during the second evaluation included age, gender, whether the participant attended school and years of schooling, marital status, household composition, alcohol consumption and smoking history, history of cancer, Parkinson’s disease, diabetes, hypertension, stroke, heart attack, head injury and bone fracture by self-report. Participants’ height and weight were also measured during the interview. BMI was derived from height and weight measurements. Blood spots on filter paper were collected from all study participants during the baseline evaluation. *APOE* genotype was determined by eluting DNA from a dried blood spot [21] followed by *HhaI* digestion of amplified products [22].

### Statistical analysis

We conduct cross-sectional analysis using GDS scores and cognitive scores collected at the second evaluation of this cohort. Mean GDS scores were compared by participants’ characteristics using analysis of variance (ANOVA) or t-tests. Significant factors in univariate association with GDS scores were included in Analyses of Covariance (ANCOVA) models. Two sets of ANCOVA models were used to examine selenium’s relationship with GDS scores. The first model included all significant covariates in addition to selenium levels as independent variables. The second ANCOVA model included a composite cognitive *z*-score obtained from the time of the GDS administration as well as all variables in the first model. To ensure robust estimation results, we also conducted nonparametric ANCOVA by using normal scores derived from the ranks of the GDS scores [23]. The composite cognitive z-score was created by using the average of standardized scores of the six cognitive tests [24,25]. To detect potential non-linear relationship between BMI, selenium levels and GDS scores, we also conducted models using tertile groups. Logistic regression models were used to investigate whether selenium levels were associated with mild or severe depression defined as GDS scores 11 or higher.

## Results

The GDS was administered to 1737 participants. Mean age in the cohort is 74.3 years (SD = 5.2), with 53.1 % being women and 61.1 % never attended school. Mean BMI was 22.3 (SD = 3.8). In Table 1, we present mean GDS scores by participants’ characteristics. Age, gender, schooling, marital status, living arrangement, BMI, alcohol, smoking, histories of stroke, heart attack, head injury, fractures and selenium levels were significantly associated with GDS scores in each separate univariate analysis. In Table 2, we present results of ANCOVA models including significant factors associated with GDS scores. Older age, living alone, lower BMI, history of stroke, heart attack, head injury and fracture were associated with higher GDS scores, while having attended school and ever drinking alcohol were associated with lower GDS scores. In this model, higher selenium level was associated with lower GDS scores while adjusting for all other significant factors in the model. In a subsequent model using selenium tertile group instead of continuous selenium levels, we also found the lowest selenium group had significant higher GDS score than those in the highest selenium group (p = 0.0063) while the medium selenium group is not significantly different from the high selenium group (p = 0.2143) (Table 3).

**Table 1:** Mean GDS scores by participants’ characteristics

	**N**	**Mean**	**SD**	**p-value**
Age group				
65-74	1002	7.68	5.83	<0.0001
75-84	652	9.02	5.86	
85+	83	9.38	5.55	
Gender				
Male	815	7.52	5.77	<0.0001
Female	922	8.92	5.87	
Ever attended school				
Yes	676	7.05	5.73	<0.0001
No	1061	9.04	5.82	
Marital status				
Married	1016	7.65	5.71	<0.0001*
Widowed	704	9.08	5.91	
Other	17	11.47	8.18	
Living arrangement^#^				
With spouse	828	7.39	5.69	<0.0001
With children	585	8.85	5.58	
Living alone	311	9.60	6.51	
Body Mass Index tertiles				
< 20.45	578	8.80	5.94	0.0025
[20.45, 23.24)	577	8.38	6.02	
≥ 23.24	582	7.62	5.57	
Ever drink alcohol				
Yes	697	7.59	5.73	<0.0001
No	1040	8.72	5.91	
Smoking				
Current smoker	553	7.72	5.89	0.0013
Former smoker	148	7.39	5.57	
Non-smoker	1036	8.68	5.85	
History of Cancer				
Yes	33	8.85	6.51	0.5634
No	1704	8.25	5.85	
History of Parkinson’s				
Yes	36	8.67	6.04	0.6774
No	1701	8.26	5.86	
History of diabetes				
Yes	75	8.25	5.83	0.5435
No	1662	8.67	6.64	
History of hypertension^				
Yes	1029	8.36	5.83	0.4141
No	708	8.13	5.91	
History of stroke^#^				
Yes	45	10.20	6.97	0.0245
No	1690	8.21	5.83	
History of heart attack				
Yes	52	12.00	6.42	>0.0001
No	1685	8.15	5.81	
History of head injury				
Yes	66	11.42	6.44	<0.0001
No	1671	8.14	5.81	
History of fracture				
Yes	30	12.47	6.00	<0.0001
No	1707	8.19	5.83	
APOE e4 carriers				
Yes	291	8.51	5.69	0.4360
No	1446	8.22	5.90	
Selenium tertiles, μg/g				0.0132
Low (<0.32)	561	8.83	5.64	
Medium [0.32, 0.47)	582	8.16	5.79	
High (≥ 0.47)	594	7.83	6.10	
Quartile groups defined by composite cognitive score				<0.0001
Q4 (75 % to 100 %)	434	5.5	4.8	
Q3 (50 % to 75 %)	434	7.7	5.7	
Q2 (25 % to 50 %)	435	9.1	5.7	
Q1 (0 to 25 %)	434	10.7	5.9	

* Comparing those married to widowed, excluding those in the “other” group.

^ Blood pressure measure ≥ 140/90 or self-reported history of hypertension.

#: 13 subjects had missing information on living arrangement and 2 subjects had missing information on the history of stroke.

**Table 2:** **Results of analysis of covariance (ANCOVA) models between selenium levels and depressive symptoms measured by GDS score adjusting for other factors (n = 1735)**^**a**^

	**Without Cognitive Score**			**With Cognitive Score**		
**Variable**	**Estimate**	**Std Err**	**p-value**	**Estimate**	**Std Err**	**p-value**
Age, years	0.10	0.03	0.0002	−0.00	0.03	0.8688
Attended school vs no school	−1.40	0.29	<0.0001	−0.41	0.29	0.1681
Live alone	1.37	0.35	0.0001	1.53	0.34	<0.0001
Ever drink alcohol	−0.97	0.29	0.0009	−0.74	0.28	0.0093
Body mass index	−0.12	0.04	0.0012	−0.05	0.04	0.1470
History of stroke	1.96	0.86	0.0223	1.62	0.83	0.0500
History of heart attack	3.31	0.80	<0.0001	3.55	0.77	<0.0001
History of head injury	2.74	0.72	0.0001	2.49	0.69	0.0003
History of fracture	3.00	1.05	0.0042	2.36	1.01	0.0190
Selenium, μg/g	−1.66	0.77	0.0321	−0.93	0.75	0.2143
Composite cognitive *z-*score				−0.38	0.20	<0.0001

^a^ Two subjects had missing information on history of stroke.

**Table 3:** **Results of analysis of covariance (ANCOVA) models between selenium tertile groups and depressive symptoms measured by GDS score adjusting for other factors (n = 1735)**^**a**^

	**Without Cognitive Score**			**With Cognitive Score**		
**Variable**	**Estimate**	**Std Err**	**p-value**	**Estimate**	**Std Err**	**p-value**
Age, years	0.10	0.03	0.0002	0.01	0.03	0.6132
Attended school vs no school	−1.39	0.29	<0.0001	−0.49	0.29	0.0932
Live alone	1.35	0.36	0.0002	1.47	0.34	<0.0001
Ever drink alcohol	−1.03	0.29	0.0005	−0.74	0.29	0.0095
Body mass index	−0.12	0.04	0.0016	−0.05	0.04	0.1311
History of stroke	1.98	0.86	0.0209	1.73	0.83	0.0364
History of heart attack	3.34	0.80	<0.0001	3.59	0.78	<0.0001
History of head injury	2.67	0.72	0.0002	2.43	0.69	0.0005
History of fracture	2.92	1.05	0.0054	2.64	1.01	0.0091
Selenium tertile group, μg/g						
<0.32	0.96	0.35	0.0063	0.58	0.34	0.0876
[0.32, 0.47)	0.42	0.34	0.2143	0.29	0.32	0.3712
≥ 0.47	reference	--	--	reference	--	--
Cognitive score quartile groups						
Q4 (75 % to 100 %)				−4.61	0.42	<0.0001
Q3 (50 % to 75 %)				−2.64	0.39	<0.0001
Q2 (25 % to 50 %)				−1.43	0.38	0.0001
Q1 (0 to 25 %)				reference	--	--

^a^ Two subjects had missing information on history of stroke.

In a second ANCOVA model in Table 2, we included the composite cognitive *z*-score in the model to determine whether selenium’s association with GDS score is independent of cognitive function. In this model, higher cognitive scores were significantly associated with lower GDS scores. However, selenium level was no longer significantly associated with GDS scores indicating that there is little direct effect from selenium on GDS scores. In addition, selenium was significantly associated with the composite cognitive z-score (p = 0.0011) in a separate ANCOVA model with the composite cognitive *z-*score as dependent variable adjusting for the same variables as in model 2.

**Figure 1:**
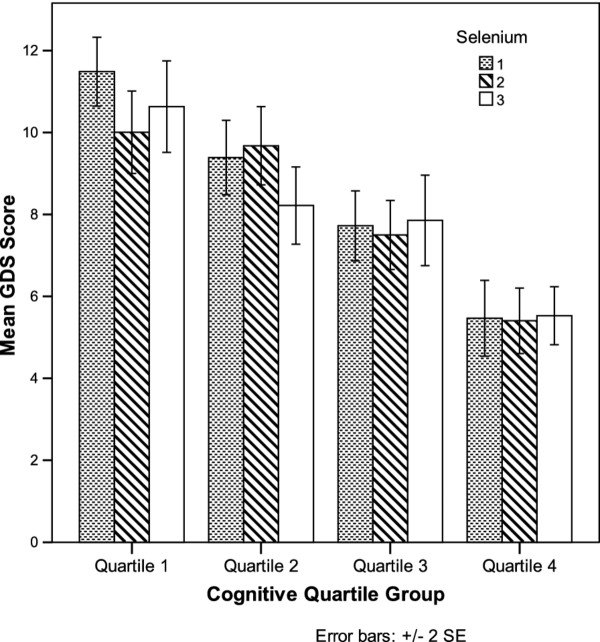
Mean GDS scores by selenium tertiles and cognitive quartile groups.

In Figure 1, mean GDS scores in each selenium tertile group were presented by quartile cognitive group. Lower GDS scores were seen in the higher cognitive group while mean GDS scores no longer show differences across selenium tertile groups. Results obtained using non-parametric ANCOVA models yielded similar results with significant selenium and GDS association in the model without cognitive score (β = −0.36, p = 0.0059) and non-significance selenium and GDS association in the model adjusting for cognitive score (β = −0.23, p = 0.0674).

Out of the 1737 individuals with GDS scores, 535 (30.8 %) met the criteria for depression (GDS ≥ 11). Mean selenium levels were 0.39 μg/g (SD = 0.18) in the depressed subjects and 0.43 μg/g (SD = 0.18) in the non-depressed subjects (p = 0.0006). Higher levels of selenium were significantly associated with lower probability of depression (OR = 0.40, p = 0.0047) in the logistic model without adjusting for cognitive function. The association between selenium levels and the probability of depression remained significant (OR = 0.50, p = 0.0331) after adjusting for cognitive function.

## Discussions

In this rural elderly Chinese cohort with an extensive range of selenium distribution, we found that lower selenium levels were significantly associated with higher depressive symptoms adjusting for demographic and medical conditions. However, when cognitive function was included as an independent variable in the model, the relationship between selenium and depressive symptoms was no longer significant, suggesting that selenium’s association with depressive symptoms is explained in part by its association with cognitive function.

Several previous publications including observational studies as well as clinical trials had examined the association between selenium levels and depression in various populations. Two studies observed that low selenium diet was associated with worsening mood [2,3]. In addition, there were studies showing that selenium supplementation or high dietary selenium was associated with improved mood with the most significant effect seen in participants with low dietary selenium [1,3,26]. However, a recent randomized trial of selenium supplementation found that a 6-month selenium supplementation had no significant effect on mood or quality of life in 501 participants age 60 to 74 from the United Kingdom [5]. The differences in study findings between the recent trial and previous results may be explained by differences in dietary selenium levels of study participants. The UK trial consisted of participants whose baseline selenium status was higher than the UK national average [5]. It is known that the brain has a unique feature in storing selenium and it also receives priority supply of selenium in times of deprivation [27]. Animal studies have shown that after generations of selenium deficiency when selenium concentrations in liver, skeletal muscle and blood dropped to below 1 % of normal level, the brain still retained 60 % of selenium as in normal controls.[7] In addition, studies have shown that plasma GP-x activity plateaus after sufficient selenium supply indicating that selenium supplementation in high selenium individuals may not provide additional benefit [28]. It is perhaps not surprising that the UK trial found no selenium effect as most of the participants would already have adequate selenium supply at baseline and additional supplementation was not able to increase mood measures. However, results of the UK trial still left open the question of whether low selenium is associated with depression in those with low dietary selenium levels at baseline.

The review of current literature suggests that long-term exposure to low selenium may be needed to impact brain function. The brain’s unique selenium metabolism may also make it more difficult to show the effect of short-term selenium exposure on brain function than on other organs.

The rural Chinese population offers an opportunity to investigate the association of long-term selenium exposure and depression by identifying populations where substantial numbers are exposed to low selenium diet for an extended length of time and comparing their depressive symptoms to those with normal selenium levels. The overwhelming majority of our study participants were life-long residents of the same towns where they were interviewed and very few take dietary supplements. Hence the ascertained selenium levels can be inferred as life-long exposure to selenium without the influence of supplements. In this stable cohort, we found a significant association between lower selenium levels and increased depressive symptoms which is consistent with previous reports in participants with low dietary selenium intake.

The mechanism underlying an association between selenium and depression is not yet clear, although published results point to the fact that selenium is important to brain functions. In animal models, selenium deficiency was shown to alter neurotransmitter turnover rate [29] and sodium selenite protected against dopamine loss [30]. In addition, the enzyme closely related to selenium activity (GP-x) protected against neuron loss [31], cell death [32], and decreased free radical generation or amyloid beta peptide [33]. In human studies, selenium supplementation was shown to reduce intractable epileptic seizures [34,35]. A recent cross-sectional study reported that whole blood GPX activity was significantly lower in depressed patients than in normal controls [36]. More research is needed to elucidate the biological mechanism for selenium’s function in the brain.

To our knowledge, no previous studies on selenium and depression have examined the role of cognitive function. Numerous studies have reported the co-existence of cognitive impairment and depression [37], although the underlying mechanism for such an association is not yet established. Some suggest that depressive symptoms are early manifestation of the dementia disorder [38], while others believe that depression represents an independent risk factor for cognitive decline [25]. We have previously reported a significant relationship between selenium level and cognitive function in the same cohort using data collected from baseline [8]. The non-significant association between selenium and depressive symptoms after adjusting for cognitive function suggests that cognitive function plays an important role in the relationship between selenium and depression.

Our study has a number of strengths. Selenium levels were measured in nail samples reflecting stable long-term exposure. The study included participants from sites with extensive range of selenium exposure. In addition, the majority of our study participants were life-long residents of the same towns where they were interviewed and the participants were known to have not taken dietary supplements. Hence the ascertained selenium levels can be inferred as life-long exposure to selenium without the influence of supplements. A number of cognitive instruments were used so that a reliable composite measure of cognitive function can be derived.

There are also important limitations in this study. Our results were based on cross-sectional analyses in the sense that cognitive function and depressive symptoms were measured at the same time. Longitudinal follow-up of this cohort would be necessary to examine the casual direction of the association between cognitive function and depressive symptoms. A second limitation is that we relied on the GDS to assess depressive symptoms rather than clinical diagnosis of specific depressive disorders, an approach adopted by many large epidemiological studies [25,38]. Further limitations include the exclusion of potentially important risk factors for depression such as disability or social support.

## Conclusions

In summary, we found that selenium level was inversely related to depressive symptoms in models without adjusting for cognition in this rural elderly Chinese cohort. However, this association appears to be accounted for by the association between selenium and cognitive function. Longitudinal follow-up of this cohort will be able to further examine the complex relationship between selenium, cognitive function and depressive symptoms.

## Competing interests

The authors report no conflicts of interest.

## Authors’ contributions

Conception and design: Drs. Gao, Jin, Hall, Liang, Unverzagt, Murrell, and Hendrie. Acquisition of data: SG, YJ, KSH, CL, JC, FWU, JRM, FM, YC, JB, PL, and HCH. Analysis and interpretation of data: SG and HCH. Drafting of the manuscript: SG, YJ, CL, and HCH. Critical revision of the manuscript for important intellectual content: SG, YJ, KSH, CL, FWU, JRM, JC, FM, YC, JB, PL, and HCH. Statistical expertise: SG. Obtaining funding: SG, YJ, KSH, CL, FWU, JRM, and HCH. Administrative, technical or material support: YJ, CL, FM, YC, PL, JB, and Matesan. Supervision: SG, YJ, CL, and HCH. All authors read and approved the final manuscript.

## Pre-publication history

The pre-publication history for this paper can be accessed here:

http://www.biomedcentral.com/1471-244X/12/72/prepub
